# 

**Published:** 1887-10

**Authors:** 


TERATOMATA—An Aetiological Study. J. Bland Sutton, M.D. .......... 295
A REVIEW OF THE MUSCLES USED IN THE CLASSIFICATION OF BIRDS. R. W. Shufeldt, M.D., U. S. A .......... 321
SCALMA. Rush S. Huidekoper, M. D. .......... 344
THE COLON OF THE HORSE—Its Diseases and Derangements. Thomas Walley, M. R. C. V. S .......... 349
CARBUNCLES IN HORSES. Daniel D. Lee, D. V. M. .......... 363
KAMRI; or, Paraplegia. R. W. Burke, V. S., A. V. D .......... 367
EDITORIAL DEPARTMENT .......... 374
CASE DEPARTMENT .......... 385
PROCEEDINGS OF VETERINARY MEDICAL SOCIETIES .......... 390
OBITUARY .......... 394
REVIEWS .......... 396

